# Dietary Magnesium Intake Ameliorates the Association Between Household Pesticide Exposure and Type 2 Diabetes: Data From NHANES, 2007–2018

**DOI:** 10.3389/fnut.2022.903493

**Published:** 2022-05-20

**Authors:** Jungao Huang, Liqin Hu, Juan Yang

**Affiliations:** ^1^Key Laboratory of Environment and Disease-Related Gene, Ministry of Education, Department of Cell Biology and Genetics, School of Basic Medical Sciences, Health Science Center, Xi'an Jiaotong University, Xi'an, China; ^2^Ganzhou Maternal and Child Health Hospital, Ganzhou, China

**Keywords:** type 2 diabetes, household pesticide exposure, dietary magnesium intake, oxidative stress, general population

## Abstract

**Aims/Hypothesis:**

This study aimed to explore whether household pesticide exposure in the general population increased the risk of developing type 2 diabetes and whether intake of dietary magnesium could lower type 2 diabetes from household pesticide exposure.

**Methods:**

For this cross-sectional study, we obtained the data of 9,187 United States adults from the National Health and Nutrition Examination Surveys, 2007–2018. Participants were subdivided into two groups based on the amount of daily dietary magnesium in the population: low group: <175 mg/day and high group: ≥175 mg/day. Using multivariable logistic regression analysis, we evaluated the relationship between pesticide exposure in the home and type 2 diabetes.

**Results:**

Compared to those unexposed at home, individuals who were exposed to pesticides in their households had a relatively higher odds ratio for type 2 diabetes (OR = 1.22, 95% CI: 1.04–1.44). The association of pesticide exposure in the home with the incidence of type 2 diabetes was different for low and high dietary magnesium groups, OR = 1.66, 95% Cl: 1.19-2.33 vs. OR = 1.1, 95% Cl: 0.92–1.32, respectively. An interaction (*P* = 0.035) between household pesticide exposure and magnesium intake, suggested that high dietary magnesium intake may reduce the risk of developing type 2 diabetes from pesticide exposure.

**Conclusions:**

Household pesticide exposure in the general population is associated with an elevated risk of type 2 diabetes. We report for the first time possible clinical relevance in that high magnesium intake may ameliorate the increased risk of type 2 diabetes from pesticide exposure.

## Introduction

Household pesticide exposure (HPE) has received much attention in the United States (US) as pesticides have been widely used to control or kill insects in homes. Moreover, there has been an increase in the global use of pesticides for agricultural purposes in recent years—estimated at 2.3 billion kg annually (1). Previous research has found an association of residential or occupational pesticide exposure with various health issues such as birth defects, asthma, some cancers, Parkinson's disease, and depression (2). Several studies also have linked pesticide exposure to type 2 diabetes, but there are limitations in the universality of these findings because the studies targeted specific groups of occupational workers or their wives (3, 4). In addition, the opposite conclusion has also been noted. Sharafi et al. (5) reported in a cross-sectional study that past exposure to pesticides was not associated with type 2 diabetes. Furthermore, a cohort study by Liu et al. indicated that even in the case of acute high dose organophosphorus exposure, the risk in the short-term of diabetes emerging is minimal (6). Studies investigating the association between HPE and type 2 diabetes involving the general population are scarce. Meanwhile, the differences in the findings of previous studies may have resulted from neglect of some potential covariates, such as high magnesium intake.

Magnesium is required for many physiological processes and acts as an important cofactor of certain enzymes in glucose and insulin metabolism (7, 8). A previous study showed that high dietary fiber and high intake of magnesium may play a beneficial role in reducing the risk of type 2 diabetes (9). A systematic review concluded that an additional 100 mg daily intake of dietary magnesium was inversely associated with the risk of type 2 diabetes (10). Similarly, another recent systematic review has shown that magnesium intake has inverse associations with type 2 diabetes. Thus the suggestion that increasing magnesium dietary patterns may be highly beneficial for populations and could perhaps be a type 2 diabetes public health prevention strategy (11). However, clinical studies on the relationship between HPE, magnesium intake, and type 2 diabetes are limited. Therefore, in this study, we investigated whether there is a positive association between HPE and type 2 diabetes in the general population, and whether dietary magnesium intake could ameliorate this association.

## Methods

### Study Population and Data Sources

The population and data were sourced from the National Health and Nutrition Examination Survey (NHANES), National Center for Health Statistics (NCHS) and the Centers for Disease Control and Prevention (CDC). They conducted these large, multistage, stratified probability surveys, which represent a non-institutionalized population of US citizens. We used the NHANES public data continuously in our analysis from 2007 to 2018 (six cycles: 2007–2008, 2009–2010, 2011–2012, 2013–2014, 2015–2016, and 2017–2018). The original protocol was approved by the Ethics Review Board of the NCHS and is available online (www.cdc.gov/nchs/nhanes/irba98.htm). All participants provided written informed consent. Those who completed the interviews and the examination, who were over the age of 19 years were included in the study. The demographic, dietary, examination, laboratory and questionnaire information were collected. Individuals with missing data on HPE, type 2 diabetes, dietary magnesium intake, and other covariates were excluded. Finally, our study included a population of 9,187 US adults.

### Diagnosis of Type 2 Diabetes

Type 2 diabetes was diagnosed according to the American Diabetes Association's criteria (12) as well as from the participants' self-reported questionnaires. Participants were classified as having type 2 diabetes using the following criteria: (1) fasting glucose (mmol/L) ≥7; (2); glycohemoglobin HbA1c (%) ≥6.5 (48 mmol/mol); (3) two-hour oral glucose tolerance test for blood glucose (mmol/L) ≥11.1; (4) random blood glucose (mmol/L) ≥11.1; (5) self-reported questionnaire data indicating long-term diabetes diagnosis by a physician, or current use of diabetes medication or insulin to lower the blood glucose level.

### HPE

HPE was defined based on the response to the item in the questionnaire: “In the past 7 days, were any chemical products used in {your/his/her} home to control fleas, roaches, ants, termites, or other insects?” The response “Yes” was defined as “household pesticide exposure,” the response “no” was defined as “pesticide unexposed,” and if the response was missing, the participant was excluded (2, 13). Moreover, we collected the data about pesticide metabolites measured in urine and a total of 3,585 participants for chlorophenols metabolites, and 1,764 participants for acephate and ethylenethio urea were available for the analysis.

### Magnesium Intake

The 24-h period data of dietary magnesium intake was collected via a dietary recall interview (midnight to midnight) in mobile examination centers. This data collection by 24-h recall interview is the most common method in large-scale surveys to determine the dietary intake and has been used by NHANES for many years, based on a consensus reached by expert groups (14). Epidemiological studies have shown that dietary intake of magnesium in the US has declined to between 175 and 225 mg/day (15). The magnesium intake was categorized into two groups, based on the lower limit value (175 mg/day).

### Potential Covariates

Our study considered age, sex, race/ethnicity, family income, obesity, total calcium level, serum cotinine level, work activity, educational level, marital status, smoking status, waist circumference, body mass index (BMI), alcohol consumption, antacids, and dietary intake such as magnesium, protein, calcium, vitamin D, and fiber as covariates. In this study we categorized race/ethnicity (as defined by NHANES) as non-Hispanic White, other Hispanic, non-Hispanic Black, Mexican American, and other races. Family income was defined by the poverty income ratio. Education level was classified as college graduate or above, high school graduate, or did not graduate from high school. Marital status was classified as married, divorced, separated, widowed, never married, and living with a partner. Participants were asked if they ever been told by a doctor or other health professional that they had weak or failing kidneys. Do not include kidney stones, bladder infections, or incontinence. A positive response for “YES,” negative for “NO” and the other for missing. Participants were asked if they had smoked more than 100 cigarettes previously. Participants who answered that they had been smoking over several days or daily when interviewed were regarded as ‘current smokers' and those not smoking currently were regarded as ‘former smokers'. Those who had not smoked at least 100 cigarettes previously, were regarded as ‘never smokers'. Alcohol consumption was based on the responses to questions as follows: “In {your/your spouses'} entire life, {have you/has he/ has she} had at least 12 drinks of any type of alcoholic beverage? In {your/spouse's} entire life, {have you/has he/has she} had at least one drink of any kind of alcohol, not counting small tastes or sips? A drink was defined as 12 oz beer, a 5 oz glass of wine, or 1.5 oz of liquor.” Participants providing a positive response, namely “YES” to any question above were classified as alcohol drinkers, while those who answered “No” were classified as non-alcohol drinkers. A BMI ≥28 was considered obese, while a BMI <28 was not. Work activity was categorized as vigorous, moderate, and light work activities. The dietary data were additionally collected for calcium, total dietary protein, vitamin D, and fiber.

### Statistical Analysis

Statistical software packages R (http://www.R-project.org, The R Foundation), Free Statistics software version 1.3 was used for data analysis (16). The sample weights were provided by NHANES (NCHS, 2007–2018). Continuous variables are represented by mean and standard deviation, and categorical variables by numbers (n) and percentages (%). The population characteristics of the pesticide exposure group and the non-exposure group were compared by performing two-tailed Student *t*-tests and Chi-square tests. Logistic regression analysis was used for evaluating the risk of type 2 diabetes from pesticide exposure. Model 1 was adjusted for no covariates and Model 2 for age, sex, race/ethnicity. In Model 3, obesity, total calcium, serum cotinine, work activity, educational level, marital status, smoking status, waist circumference, BMI, alcohol intake, antacids, kidney disease, family income, and dietary index, including fiber, protein, calcium, magnesium, and vitamin D were considered, in addition to Model 2 adjustments. The subgroup analyses were performed between the dietary magnesium intake groups. The likelihood ratio test was used to evaluate the interaction among subgroups. Odds ratios (Ors) and 95% confidence intervals (Cis) were calculated. A two-sided *P* < 0.05 was considered statistically significant.

## Results

### Baseline Characteristics of the Study Population

**Figure 1:**
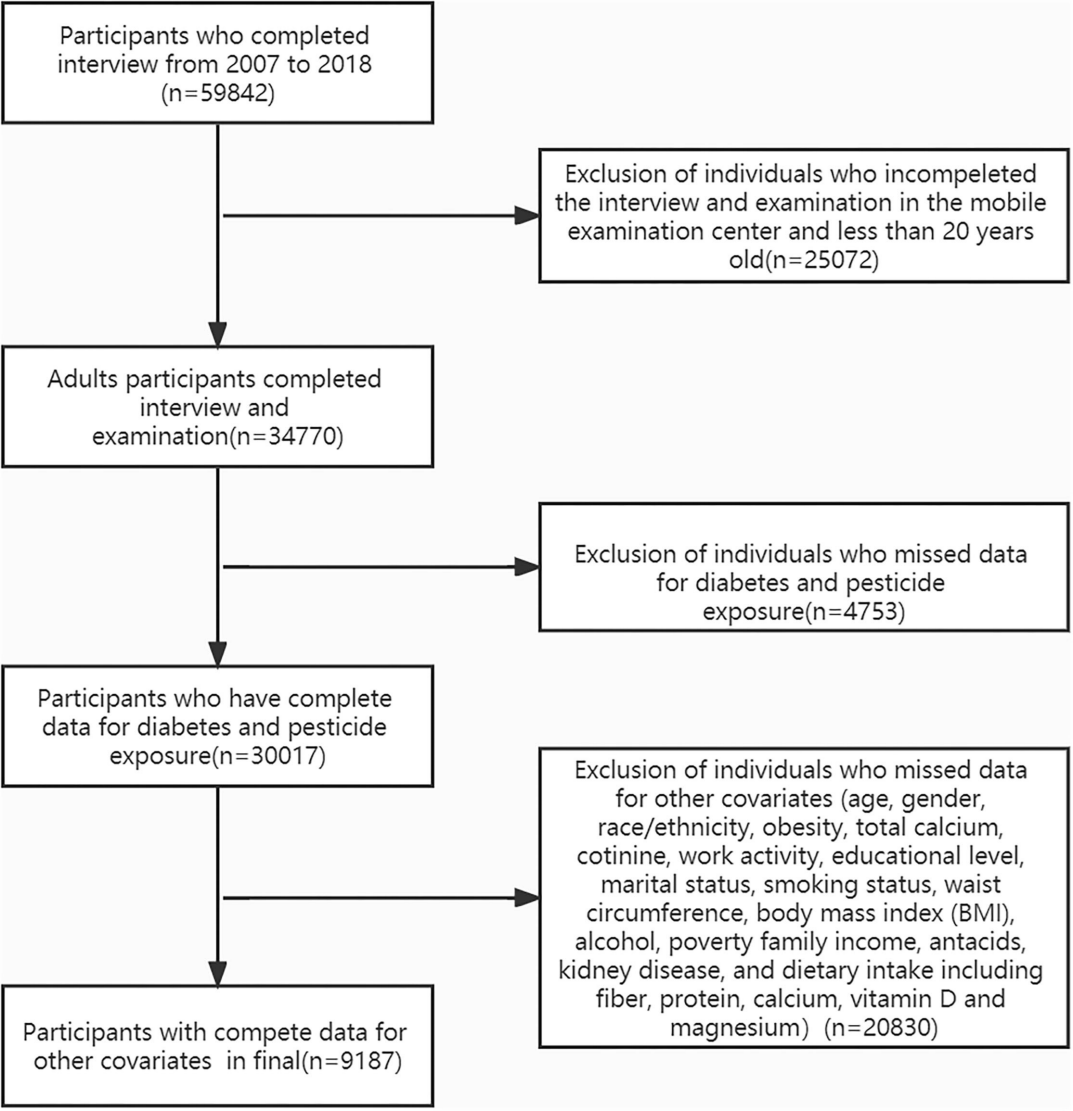
The flow chart of the study.

The flow chart of the exclusion criteria for the study population is shown in Figure 1. Of 9,187 US adults eligible for our final analysis, 911 (9.9%) individuals reported themselves exposed to pesticides in their homes. The overall mean age was 51.9 ± 17.7 years, 62.1% were female, and 31.5% had type 2 diabetes. At baseline, characteristics between the pesticide and non-pesticide exposure groups differed for age, race/ethnicity, poverty income ratio, marital status, cotinine, and type 2 diabetes. Compared with those in the non-exposure group, those in the exposed group were more likely to be older and had a lower income. The prevalence of type 2 diabetes in the non-exposed group and exposed groups were 30.8 and 37.2%, respectively (Table 1).

**Table 1:** Baseline characteristics of the study sample.

**Variables**	**Total** **(*n* = 9,187)**	**Pesticide unexposed** **(*n* = 8,276)**	**Pesticide exposed** **(*n* = 911)**	***P*** **-value**
**Gender**, ***n*** **(%)**				0.898
Female	5,705 (62.1)	5,137 (62.1)	568 (62.3)	
Male	3,482 (37.9)	3,139 (37.9)	343 (37.7)	
**Age (years)**	51.9 ± 17.7	51.7 ± 17.7	53.7 ± 17.4	0.002
**Race/Ethnicity**, ***n*** **(%)**				<0.001
Mexican American	1,404 (15.3)	1,252 (15.1)	152 (16.7)	
Non-Hispanic white	3,314 (36.1)	3,014 (36.4)	300 (32.9)	
Non-Hispanic black	2,081 (22.7)	1,835 (22.2)	246 (27)	
Other Hispanic	974 (10.6)	869 (10.5)	105 (11.5)	
Other races	1,414 (15.4)	1,306 (15.8)	108 (11.9)	
**Poverty income ratio, Median (IQR)**	1.9 (1.1, 3.6)	2.0 (1.1, 3.7)	1.6 (1.0, 3.0)	<0.001
**Education level**, ***n*** **(%)**				0.149
Did not graduate from high school	2,266 (24.7)	2,021 (24.4)	245 (26.9)	
Graduated from high school	2,181 (23.7)	1,959 (23.7)	222 (24.4)	
College education or above	4,740 (51.6)	4,296 (51.9)	444 (48.7)	
**Marital status**, ***n*** **(%)**				0.016
Married	4,777 (52.0)	4,341 (52.5)	436 (47.9)	
Widowed	933 (10.2)	829 (10)	104 (11.4)	
Separated	306 (3.3)	279 (3.4)	27 (3)	
Never married	1,537 (16.7)	1,390 (16.8)	147 (16.1)	
Divorced	991 (10.8)	870 (10.5)	121 (13.3)	
Living with partner	643 (7.0)	567 (6.9)	76 (8.3)	
**BMI (kg/m2)**	29.8 ± 7.2	29.7 ± 7.2	30.1 ± 7.4	0.131
**Waist circumference, Median (IQR)**	99.0 (88.3, 110.5)	99.0 (88.2, 110.3)	99.5 (89.8, 112.0)	0.133
**Alcohol, n (%)**				0.768
NO	3,111 (33.9)	2,798 (33.8)	313 (34.4)	
YES	6,076 (66.1)	5,478 (66.2)	598 (65.6)	
**Obesity**, ***n*** **(%)**				0.283
No	4,173 (45.4)	3,775 (45.6)	398 (43.7)	
Yes	5,014 (54.6)	4,501 (54.4)	513 (56.3)	
**T2D**, ***n*** **(%)**				<0.001
No	6,296 (68.5)	5,724 (69.2)	572 (62.8)	
Yes	2,891 (31.5)	2,552 (30.8)	339 (37.2)	
**Smoking status**, ***n*** **(%)**				0.085
Never smoker	6,272 (68.3)	5,679 (68.6)	593 (65.1)	
Former smoker	1,694 (18.4)	1,513 (18.3)	181 (19.9)	
Current smoker	1,221 (13.3)	1,084 (13.1)	137 (15)	
**Work_activity**, ***n*** **(%)**				0.245
Light work activity	5,441 (59.2)	4,917 (59.4)	524 (57.5)	
Moderate work activity	2,028 (22.1)	1,830 (22.1)	198 (21.7)	
Vigorous work activity	1,718 (18.7)	1,529 (18.5)	189 (20.7)	
**Cotinine (ng/ml), Median (IQR)**	0.0 (0.0, 0.3)	0.0 (0.0, 0.2)	0.0 (0.0, 0.5)	<0.001
**Kidneys diseases**, ***n*** **(%)**				0.842
NO	8,840 (96.2)	7,965 (96.2)	875 (96)	
YES	347 (3.8)	311 (3.8)	36 (4)	
**Total calcium (mmol/L), Median(IQR)**	2.3 (2.3, 2.4)	2.3 (2.3, 2.4)	2.3 (2.3, 2.4)	0.476
**Dietary factors**				
Magnesium (mg), Median (IQR)	253.0 (184.0, 344.0)	252.0 (184.0, 343.0)	257.0 (181.5, 349.5)	0.702
Protein (gm), Median (IQR)	67.8 (48.3, 94.3)	67.5 (48.3, 94.0)	69.5 (47.8, 96.2)	0.494
Fiber (gm), Median (IQR)	14.2 (9.2, 20.9)	14.2 (9.2, 20.8)	14.2 (9.0, 21.5)	0.997
Calcium (mg), Median (IQR)	764.0 (494.0, 1123.0)	765.0 (495.0, 1121.0)	743.0 (480.0, 1140.0)	0.606
Vitamin D (mg), Median (IQR)	2.9 (1.1, 5.7)	2.9 (1.1, 5.7)	2.9 (1.1, 5.7)	0.865
**Antacids**, ***n*** **(%)**				0.241
No	8,879 (96.6)	7,992 (96.6)	887 (97.4)	
Yes	308 (3.4)	284 (3.4)	24 (2.6)	

### Association of Covariates and Type 2 Diabetes Risk

From the univariate analysis results, it was revealed that age, sex, race/ethnicity, obesity, cotinine, work activity, educational level, marital status, smoking status, waist circumference, family income, BMI, alcohol, kidney disease, and some dietary indices such as calcium, protein, and magnesium were associated with type 2 diabetes (Table 2).

**Table 2:** Association of covariates and type 2 diabetes risk.

**Variable**	**OR_95%CI**	***P*** **-value**
Age	1.04 (1.04–1.05)	<0.001
**Gender**, ***n*** **(%)**		
Female	1 (reference)	
Male	1.09 (1–1.19)	0.062
**Race/ethnicity**, ***n*** **(%)**		
Mexican American	1 (reference)	
Non-Hispanic white	0.75 (0.66–0.85)	<0.001
Non-Hispanic black	0.77 (0.67–0.89)	<0.001
Other Hispanic	0.9 (0.75–1.06)	0.206
Other races	0.69 (0.59–0.81)	<0.001
PIR	0.93 (0.91–0.96)	<0.001
**Education level**		
Did not graduate from high school	1 (reference)	
Graduated from high school	0.63 (0.56–0.71)	<0.001
College education or above	0.53 (0.48–0.59)	<0.001
**Marital status**		
Married	1 (reference)	
Widowed	1.83 (1.59–2.11)	<0.001
Separated	1.06 (0.83–1.35)	0.658
Never married	0.5 (0.43–0.57)	<0.001
Divorced	1.11 (0.96–1.28)	0.17
Living with partner	0.54 (0.44–0.66)	<0.001
BMI	1.06 (1.06–1.07)	<0.001
Waist circumference	1.04 (1.03–1.04)	<0.001
**Alcohol**		
NO	1 (reference)	
YES	0.87 (0.8–0.96)	0.004
Cotinine	1 (1)	0.009
**Kidney disease**		
No	1 (reference)	
YES	2.78 (2.24–3.46)	<0.001
Total calcium	1.38 (0.87–2.21)	0.171
Magnesium	1 (1)	<0.001
Protein	1 (1)	<0.001
Fiber	1 (0.99–1)	0.127
Calcium	1 (1)	<0.001
VitaminD	1 (0.99–1.01)	0.976
**Antacids**		
No	1 (reference)	
YES	1.08 (0.85–1.38)	0.526
**Obesity**		
NO	1 (reference)	
YES	2.24 (2.05–2.46)	<0.001
**Smoking status**		
Never smoker	1 (reference)	
Former smoker	1.6 (1.43–1.79)	<0.001
Current smoker	0.82 (0.71–0.94)	0.005
**Work_activity**		
Light work activity	1 (reference)	
Moderate work activity	0.8 (0.71–0.89)	<0.001
Vigorous work activity	0.64 (0.56–0.72)	<0.001

### Associations Between Pesticide Exposure in Home and Type 2 Diabetes Risk

Table 3 shows that when compared to the unexposed group, pesticide-exposed participants had a higher OR (OR = 1.33, 95% CI: 1.15–1.53) for type 2 diabetes in Model 1. When adjusted for sex, age, and race/ethnicity in Model 2, the OR was 1.24 (95% CI:1.07–1.44). Further, based on Model 2 and additionally adjusted for obesity, total calcium level, cotinine level, work activity, educational level, marital status, smoking status, waist circumference, BMI, alcohol, antacids, kidney disease, family income, and dietary intake in Model 3, the OR was 1.22 (95% CI: 1.04–1.44). Among associations between pesticide metabolites and type 2 diabetes risk, we found that O-Phenyl phenol was significantly associated with type 2 diabetes risk (OR = 1.20, 95% CI: 1.00–1.44, *P* = 0.048) (Supplementary Table S1).

**Table 3:** The associations between pesticide exposure in home and type 2 diabetes risk.

	***N*** **(%)**	**OR**	**95%CI**	***P*** **-value**
**Model 1**				
Pesticide unexposed	8,276 (30.8)	Reference		
Pesticide exposed	911 (37.2)	1.33	1.15–1.53	<0.001
**Model 2**				
Pesticide unexposed	8,276(30.8)	Reference		
Pesticide exposed	911 (37.2)	1.24	1.07–1.44	0.005
**Model 3**				
Pesticide unexposed	8,276 (30.8)	Reference		
Pesticide exposed	911 (37.2)	1.22	1.04–1.44	0.013

*OR, odds ratio; CI, confidence intervals*.

*Model 1 unadjusted*.

*Model 2 adjusted for age, gender, and race/ethnicity*.

*Model 3 adjusted for age, gender, race/ethnicity, obesity, total calcium, cotinine, work activity, educational level, marital status, smoking status, waist circumference, BMI, poverty family income, alcohol, antacids, kidney disease, and dietary intake including fiber, protein, calcium, vitamin D and magnesium*.

### Dietary Magnesium Intake Ameliorates the Association Between HPE and Risk of Type 2 Diabetes

An interaction was found between magnesium intake and HPE with respect to type 2 diabetes (interaction likelihood ratio test: *P* = 0.035) (Table 4). Stratified analysis by magnesium intake was performed. In one group (high magnesium intake: ≥175 mg/day), these rates of type 2 diabetes in the pesticide exposed group and non-exposure group were 34.9 and 29.9%, respectively. Adjusting for other confounders, the results of the multivariable logistic regression showed that there was no positive association between HPE and type 2 diabetes (OR = 1.1, 95% CI: 0.92–1.32, *P* = 0.297). However, in the other group (low magnesium intake: <175 mg/day), The rate of type 2 diabetes in the pesticide exposed group and non-exposed group was 44.7 and 34.0%, respectively. When adjusting for other confounders in the populations with a low magnesium diet, pesticide exposure increased the risk of type 2 diabetes by 66% compared with no exposure (OR = 1.66, 95% CI: 1.19–2.33, *P* = 0.003).

**Table 4:** The associations between pesticide exposure in home and type 2 diabetes risk by dietary magnesium intake.

**Subgroup**	***N* (%)**	**OR**	**95%CI**	***P*-value**	***P* for interaction**
**Magnesium intake (<175 mg/d)**					0.035
Pesticide unexposed	1,815 (34.0)	Reference			
Pesticide exposed	217 (44.7)	1.66	1.19–2.33	0.003	
**Magnesium intake (≥175 mg/d)**					
Pesticide unexposed	6,461 (29.9)	Reference			
Pesticide exposed	694 (34.9)	1.1	0.92–1.32	0.297	

*OR, odds ratio; CI, confidence intervals*.

*Adjusted for age, gender, race/ethnicity, obesity, total calcium, cotinine, work activity, educational level, marital status, smoking status, waist circumference, BMI, poverty family income, alcohol, antacids, kidney disease, and dietary intake including fiber, protein, calcium, vitamin D and magnesium*.

## Discussion

This study, which used a nationally representative sample of the general US population, found a significant association between pesticide use in the home and the risk of type 2 diabetes. We have reported for the first time the interaction between daily intake of magnesium and pesticide exposure in the home with respect to the risk of type 2 diabetes. This suggests that dietary magnesium intake may ameliorate the detrimental effect of pesticide use in the home on type 2 diabetes.

Many studies have linked pesticide exposure to diabetes in occupational populations. Juntarawijit et al. (1) reported that the occurrence of diabetes among Thai farmers was associated with pesticide exposure. Saldana et al. (17) found that exposure to agricultural pesticides early in pregnancy may increase the likelihood of developing gestational diabetes. The Agricultural Health Study shows that long-term exposure to pesticides containing organochlorine and organophosphorus may increase the risk of developing diabetes (4). Raafat et al. (18) also found a strong association between blood levels of organophosphorus insecticides and insulin resistance among farmers. In addition, results from a cross-sectional study showed that farmers with occupational exposure to organophosphorus pesticides were prone to neuropsychological disease and diabetes (19). Supportive of our study, results from a multicenter cohort study reveal that beta-cell function impairment in subjects may be attributable to pesticide exposure (20). Moreover, a systematic review, including 22 studies, on pesticide exposure and type 2 diabetes found a mean OR of 1.58 (95% CI:1.32–1.90) (21). Our study supports and extends the findings of previous studies showing that even routine pesticide use in home is associated with the risk of developing type 2 diabetes. Although the underlying mechanisms of the relationship between pesticide exposure and type 2 diabetes have not been fully elucidated, it is believed that pesticides demonstrate their toxic influence through cellular oxidative stress, disrupting the hormonal and neuronal status of our bodies (22).

However, in a population-based, prospective cohort, Magliano et al. did not observe any relationship between exposure to 22 kinds of organic pollutants and diabetes (23). Similarly, in one cross-sectional study of 9,088 participants, the data did not show a relationship between pesticide use and the risk of developing type 2 diabetes (5). The reason for these contrasting results in the above studies may be that covariates or important potential confounders were not considered, taking dietary magnesium intake for example. A recent randomized controlled trial has shown that oral magnesium supplementation could affect blood sugar level in patients with type 2 diabetes (24). Studies have concluded that intake of foods rich in magnesium reduces the risk of type 2 diabetes (10, 25), and a long-term prospective study reports that magnesium intake is inversely associated with diabetes incidence (26). Moreover, a cross-sectional study has shown that individuals in the US with a high magnesium intake were found in individuals that were male, better educated, and had a higher income (27). Interestingly, a nested case-control study conducted in urban India found that there was no positive association between a pesticide metabolite and type 2 diabetes incidence (28). Another cross-sectional study in urban Nepali individuals found a negative association between pesticide exposure and diabetes (29). Conversely, a study conducted in a rural population in Korea found that pesticide exposure was associated with diabetes (30). The omission of the potentially important covariate of high magnesium intake may account for the negative associations found in studies conducted in the rich, well-educated, urban population.

As a cofactor of over 600 enzymes, magnesium is required in hundreds of essential biochemical reactions (31). For instance, magnesium, as a cofactor of glutathione reductase, has several important functions, such as antioxidant defense and xenobiotic detoxification (32). It is also well-established that magnesium has a protective effect against oxidative stress and reduces oxidative damage mediated by free radicals (33). Various experimental evidence explains magnesium's antioxidant and defensive role against some toxic substances (34, 35), which may imply the importance of magnesium in restoring oxidative damage provoked by pesticides. Furthermore, Shafeeq and Mahboob's study in a rat model found that magnesium supplementation ameliorated the toxicity of 2,4-dichlorophenoxyacetic acid (36). As is known, magnesium sulfate is used to reverse pesticide poisoning. Therefore, since magnesium sulfate was used in the study conducted by Liu et al., it may not be accurate to conclude that in the case of acute high dose organophosphorus exposure, the risk of short-term emerging diabetes is minimal due to the neglect of the role of magnesium (6).

There are some limitations to our study. First, the cross-sectional nature of the present study precludes the inference of a cause-effect relationship. Second, in the NHANES database, detailed information of household pesticides are not available, like the type, intensity, frequency, and duration of exposure, which may have various effects on type 2 diabetes. Third, recall bias on pesticide use and 24-h dietary data may be present in the interview. Fourth, although the method of multi-stage stratified probability design was used, the participants included in NHANES were American and may not be well-representative of other countries or geographical regions. Given the above limitations, well-designed multicenter controlled trials are needed to validate our findings.

## Conclusions

In summary, our study provides further evidence that the association exists between HPE in the general population and an increased risk of type 2 diabetes. We report for the first time that the interaction exists between daily magnesium intake and HPE on developing type 2 diabetes risk, indicating that high dietary magnesium intake might ameliorate the negative effect of pesticide use on the risk of type 2 diabetes. Although this study raises caution about the use of pesticides in the home because of the increased risk of type 2 diabetes triggered by its chronic toxicity, more evidence is needed from randomized controlled studies, and further experimental studies are essential to elucidate the relationship among pesticides, magnesium, and type 2 diabetes.

## Data Availability Statement

The original contributions presented in the study are included in the article/Supplementary Material, further inquiries can be directed to the corresponding author/s.

## Ethics Statement

The original protocol was approved by the Ethics Review Board of the NCHS and is available online (https://www.cdc.gov/nchs/nhanes/irba98.htm). All participants provided written informed consent.

## Author Contributions

JH drafted manuscript. LH collected clinical data. JY reviewed data analyses. All authors read and approved the final manuscript.

## Funding

The Scientific Research and Sharing Platform Construction Project of Shaanxi Province (Grant Number: 2022PT-07).

## Conflict of Interest

The authors declare that the research was conducted in the absence of any commercial or financial relationships that could be construed as a potential conflict of interest.

## Publisher's Note

All claims expressed in this article are solely those of the authors and do not necessarily represent those of their affiliated organizations, or those of the publisher, the editors and the reviewers. Any product that may be evaluated in this article, or claim that may be made by its manufacturer, is not guaranteed or endorsed by the publisher.
