# Supplementary material for: Dietary Magnesium Intake Ameliorates the Association Between Household Pesticide Exposure and Type 2 Diabetes: Data From NHANES, 2007–2018
Source: Front Nutr. 2022 May 20;9:903493. doi: 10.3389/fnut.2022.903493 (PMC9165529; doi:10.3389/fnut.2022.903493)
Supplement: Supplementary file 1 [file Table_1.DOC]

Supplementary STable 1

The associations between pesticide metabolites and T2D risk.

OR = odds ratio, CI = confidence intervals.

|  | OR | 95%CI | P-value |
| --- | --- | --- | --- |
| 2,4-dichlorophenol (ug/L) | 1.00 | 1.00-1.01 | 0.523 |
| 2,5-dichlorophenol (ug/L) | 1.00 | 1.00-1.00 | 0.481 |
| 2,4,5-trichlorophenol (ug/L) | 1.05 | 0.60-1.83 | 0.860 |
| 2,4,6-trichlorophenol (ug/L) | 1.03 | 0.96-1.12 | 0.403 |
| O-Phenyl phenol (ug/L) | 1.20 | 1.00-1.44 | 0.048 |
| Acephate (ug/L) | 0.63 | 0.32-1.25 | 0.186 |
| Ethylenethio urea (ug/L) | 0.93 | 0.52-1.66 | 0.798 |

Adjusted for age, gender, race/ethnicity, poverty family income, educational level, marital status, BMI, obesity, total calcium, work activity, smoking status, alcohol, and dietary intake including fiber, protein, vitamin D and magnesium.
